# Effect of physical activity on paired-pulse suppression in the somatosensory cortex after tibial nerve stimulation

**DOI:** 10.1007/s00221-026-07340-8

**Published:** 2026-06-25

**Authors:** Steven Röhner, Tom Maudrich, Rouven Kenville, Patrick Ragert

**Affiliations:** 1 Department of Movement Neuroscience, Faculty of Sport Science, University of Leipzig, Jahnallee 59, 04109 Leipzig, Germany; 2 Department of Neurology, Max Planck Institute for Human Cognitive and Brain Sciences, Leipzig, Germany

**Keywords:** Paired-pulse suppression (PPS), Somatosensory cortex, Somatosensory evoked potentials (SEPs), Intracortical inhibition, Tibial nerve stimulation, Physical activity

## Abstract

Paired-pulse somatosensory evoked potentials (ppSEPs) offer a robust, non-invasive measure of inhibitory interactions in the human somatosensory cortex. The impact of long-term habitual physical activity on gating mechanisms remains poorly understood. This study quantified whether regular physical activity over extended periods modulates paired-pulse suppression (PPS) after tibial nerve (TN) stimulation and whether PPS relates to weekly training volume. Fifty-nine healthy participants (29 female; 22.4 ± 3.9 years) were analyzed. Physical activity over the previous two years was assessed by questionnaire and participants were classified as physically active (PA, n = 38) if they reported more than 1.25 h/week of regular training during this period. All others were classified as non-active (NA, n = 21) participants. Single-pulse (spSEP) and ppSEPs were recorded bilaterally after suprathreshold TN stimulation using a Cz’–Fz montage. Across all participants tested, significant PPS was observed for N30/P40, P40/N50 and N50/P60 ppSEP amplitudes after both left and right TN stimulation. However, PPS and spSEP did not differ between PA and NA. PPS in right N30/P40 correlated negatively with weekly training hours in PA. No such associations were found for P40/N50 or N50/P60, nor for left-sided PPS measures. Long-term physical activity does not seem to modulate SEP components or PPS after TN stimulation. However, training volume within PA was associated with PPS of the N30/P40 amplitude after right TN stimulation, indicating a potential selective dose-dependent modulation. Longitudinal studies are needed to establish causality, explain side-specific effects, and determine the behavioral relevance for sensorimotor performance.

## Introduction

Paired-pulse somatosensory evoked potentials (ppSEPs) provide a robust and reliable neurophysiological approach for assessing inhibitory interactions within the human somatosensory cortex (Tinazzi et al. 1998; Höffken et al. 2010). By delivering two closely spaced suprathreshold electrical stimuli to a peripheral nerve, paired-pulse suppression (PPS) of specific SEP amplitudes has been identified. In fact, the majority of ppSEP studies stimulated the median nerve (MN) of the upper limb. This suppression is regarded as an indicator for intracortical inhibition which seems to depend on GABA_A_-mediated mechanisms (Stude et al. 2016). PPS of SEP amplitudes such as the N20/P25 is most prominently observed after median nerve stimulation at interstimulus intervals (ISIs) between 20 and 40 ms (REFS) and seems to depend on specific parameter settings such as the repetition rate of peripheral nerve stimulation. Other parameters such as the stimulation intensity do not seem to influence the outcome of PPS (Gatica Tossi et al. 2013; Höffken et al. 2013). PPS is typically quantified by calculating the ratio of SEP amplitudes (Ragert et al. 2004) or by subtracting ppSEP amplitudes from a spSEP condition (Kenville et al. 2025). Apart from stimulating the median nerve, ppSEPs after tibial nerve (TN) stimulation with an ISI of 60 ms has been shown to suppress the P40/N50 amplitude which is assumed to be of cortical origin (Miura et al. 2003) since it is most likely generated within the primary somatosensory cortex (S1) (Bettmann et al. 2020).

ppSEP paradigms have emerged as a highly informative neurophysiological tool for studying adaptive and maladaptive plasticity within and between somatosensory cortices (Ragert et al. 2011; Höffken et al. 2013). For example, Lenz and colleagues found that PPS after MN stimulation significantly declined with age, indicating that elderly participants show reduced intracortical inhibition which also impacted perceptual abilities (Lenz et al. 2012). Likewise, studies have shown that PPS can be modulated through experience-dependent mechanisms such as motor and perceptual learning (Höffken et al. 2007; Ragert et al. 2008; Saito et al. 2019; Pham et al. 2022) or by means of non-invasive brain stimulation protocols (Ragert et al. 2008). In clinical populations, altered ppSEP responses have been identified in several neurological disorders such as amyotrophic lateral sclerosis, neuropathic pain, dystonia, migraine, Parkinson’s disease and multiple sclerosis, indicating that deficits in inhibitory control may represent a neurophysiological hallmark of these disorders (Namerow and Etemadi 1970; Nakashima et al. 1992; Frasson et al. 2001; Valeriani et al. 2005; Lenz et al. 2011; Höffken et al. 2019). Consequently, ppSEPs have become a valuable non-invasive technique for investigating inhibitory mechanisms in both basic neuroscience and clinical neurophysiology, offering insights into how the brain filters, gates, and prioritizes sensory information under various physiological and pathological conditions.

Apart from specific stimulation parameters and factors such as age or disease, several other studies showed that PPS is significantly modulated by other determinants such as gender (Anazawa et al. 2023), or alcohol intake (Mochizuki et al. 2006). Even though there is convincing evidence that exercise and skill learning/training modulates functional brain processing (Herholz and Zatorre 2012; Sousa Fernandes et al. 2020) and is capable of inducing structural neuroplasticity (Taubert et al. 2012), little is known about its effects on PPS in the somatosensory system.

By means of transcranial magnetic stimulation (TMS), for example, it has been shown that experts in a specific skill such as athletes as well as musicians show altered brain processing within (Nordstrom and Butler 2002; Giboin et al. 2021) and between primary motor cortices (M1) (Ridding et al. 2000; Tanel et al. 2025). However, the directionality of reported changes in inhibition is divergent. While studies in musicians point towards decreased inhibition within and across M1 (Ridding et al. 2000; Izbicki et al. 2023), some studies in athletes indicated that motor cortical inhibition measured with short- and long-interval intracortical inhibition was increased in athletes (Dai et al. 2016). On the other hand, acute moderate-intensity (aerobic) exercise or skill training has been shown to decreased motor cortical inhibition (Perez et al. 2004; Opie and Semmler 2019) and PPS in S1, which was associated with altered temporal discrimination abilities (Yamazaki et al. 2020).

Hence, the aim of the present study was to quantify if regular exercise over longer time periods is capable of modulating PPS after TN stimulation. We hypothesized that PPS is less pronounced in physically active participants and that the amount of individual exercise (hours per week) is linked with the observed amount of PPS.

## Material and methods

### Ethical statement

The study was approved by the local Ethics Committee of Leipzig University (ref.no. 096/20-ek). All participants gave their written informed consent to participate in the study, in accordance with the Declaration of Helsinki.

### Participants

61 healthy participants (29 female) were enrolled in the present study (age: 22.5 ± 4.0, M ± SD). Physical activity over the past two years was assessed using a questionnaire. 39 participant reported that they regularly engage in specific organized sports such as: soccer (n = 13), volleyball (n = 5), handball (n = 3), fencing (n = 1), javelin throwing (n = 1), tennis (n = 2), water polo (n = 1), powerlifting (n = 1), gymnastics (n = 2), karate (n = 1), canoe (n = 2), dancing (n = 1), kickboxing (n = 1), cycling (n = 1), running (n = 1) and triathlon (n = 1). Based on these data, participants were classified into two groups: a physically active group (PA; ≥ 1.25 h of training per week) and non-active group (NA). The classification was based on the World Health Organization (WHO) recommendation for physical activity (≥ 75 min/week) and on previous studies using WHO-based physical activity classifications (Bull et al. 2020; Dos Santos et al. 2022; Ma et al. 2024). Participants in PA reported to engage in regular organized physical activity for 6.8 ± 5 h/week. Participants in NA did not engage in regular organized physical activity but still reported 0.2 ± 0.4 h/week. Two participants had to be excluded from the overall analysis. One participant showed abnormal ppSEP values exceeding 1.5 times the interquartile range of the respective group data set. Another participant was excluded from analyses due to faulty measurements. The remaining sample of 59 participants (29 female; age: 22.4 ± 3.9) was used for further analyses (PA: n = 38, 19 female; NA: n = 21, 10 female).

### Single- and paired-pulse SEP recordings

The following steps for spSEP and ppSEP recordings and analyses were previously outlined in a publication by our research group (Kenville et al. 2025). For spSEP and ppSEP recordings, two Ag–AgCl cup electrodes were placed at Cz’ and Fz according to the international 10–20 system, with Cz’ positioned 2 cm posterior to Cz. In this study, we used a Nihon Kohden Neuropack X1 system (Nihon Kohden Corp., Japan) to record and analyze spSEPs and ppSEPs following left and right TN stimulation. To reduce impedance, the areas around Cz’ and Fz were initially prepared with an abrasive paste (OneStep AbrasivePlus Gel). Subsequently, Ag–AgCl cup electrodes were placed over Cz’ (recording electrode) and Fz (reference electrode) using a conductive paste. Impedance was checked and kept below 10 kΩ throughout the experiment. Participants then positioned themselves supine on a standard massage table. TN stimulation was applied using a block electrode placed below the medial malleolus. Participants were instructed to relax as much as possible during both the preparation and recording phase. SEP recordings were sampled at 5120 Hz with an online bandpass filter set between 5 and 1500 Hz. To ensure reliable SEP signals, the stimulation intensity was set to 2 mA above the motor threshold, which is the minimum intensity required to elicit a motor response in the innervated muscles. Two types of stimulations, spSEP and ppSEP, were applied to each leg for each participant. For spSEPs, a total number of 400 square-wave pulses (0.2 ms) were delivered at 3 Hz, while 400 paired square-wave pulses (0.2 ms with a 60 ms ISI) were delivered at 3 Hz for ppSEPs. The order of stimulation type and the TN being stimulated were randomized across participants to avoid potential sequence order effects. Averaged SEP traces for each participant were used to measure the peak-to-peak amplitudes of presumed cortical responses such as N30/P40, P40/N50, and N50/P60 amplitudes, which were then used for statistical analyses. To estimate ppSEP responses, spSEP traces were subtracted from ppSEP traces. PPS was subsequently calculated for the N30/P40, P40/N50, and N50/P60 amplitudes by comparing the amplitude differences of the subtracted trace and the spSEP trace. Here, a PPS value greater than 1 indicates facilitation of SEP amplitudes, while a value less than 1 indicates inhibition.

### Statistical analysis

Statistical analyses were carried out using RStudio (version 2025.09.1 + 401). Descriptive statistics (means and standard deviations) were calculated for all SEP amplitudes for each TN stimulation. Furthermore, stimulation intensity was compared between left and right TN stimulation, with group (PA vs. NA) as between-subject factor, using an aligned rank transform ANOVA (ARTool, version 0.11.2). A single value in the spSEP P40/N50 amplitude in the PA group (left TN stimulation) was missing and was therefore excluded for all analyses involving this component. Normality was assessed using Shapiro–Wilk tests (α = 0.05), and homogeneity of variances was examined using Levene`s tests. Because several PPS measures deviated from normality, non-parametric tests were used to assess spSEP and ppSEP components. For ppSEPs the reference one-sided one-sample Wilcoxon signed-rank tests (reference value = 1) were conducted separately for each SEP component and TN stimulation. To test spSEPs and PPS between PA and NA, aligned rank transform ANOVA were performed with the following within-subject factors TN stimulation (left vs. right) and components (N30/P40, P40/N50, N50/P60). To investigate potential associations between spSEPs and PPS and amount of physical activity time per week, spearman correlation analyses were performed for each combination of TN stimulation and spSEP/ PPS. Therefore, to assess the direct effect of physical activity, correlation analyses were conducted only in PA. To control for multiple testing, Holm-Bonferroni correction was applied.

## Results

### TN stimulation intensity

Stimulation intensitydid not differ between PA and NA (*F*(1, 57) = 3.40, *p* = 0.070, PA: 11.7 ± 2.4 mA, NA: 12.1 ± 2.0 mA) or between left and right TN stimulation (*F(*1, 57) = 1.36, *p* = 0.249, left: 11.5 ± 3.0 mA, right: 11.8 ± 2.2 mA). Values are presented as mean ± SD.

### spSEP amplitudes

**Fig. 1:**
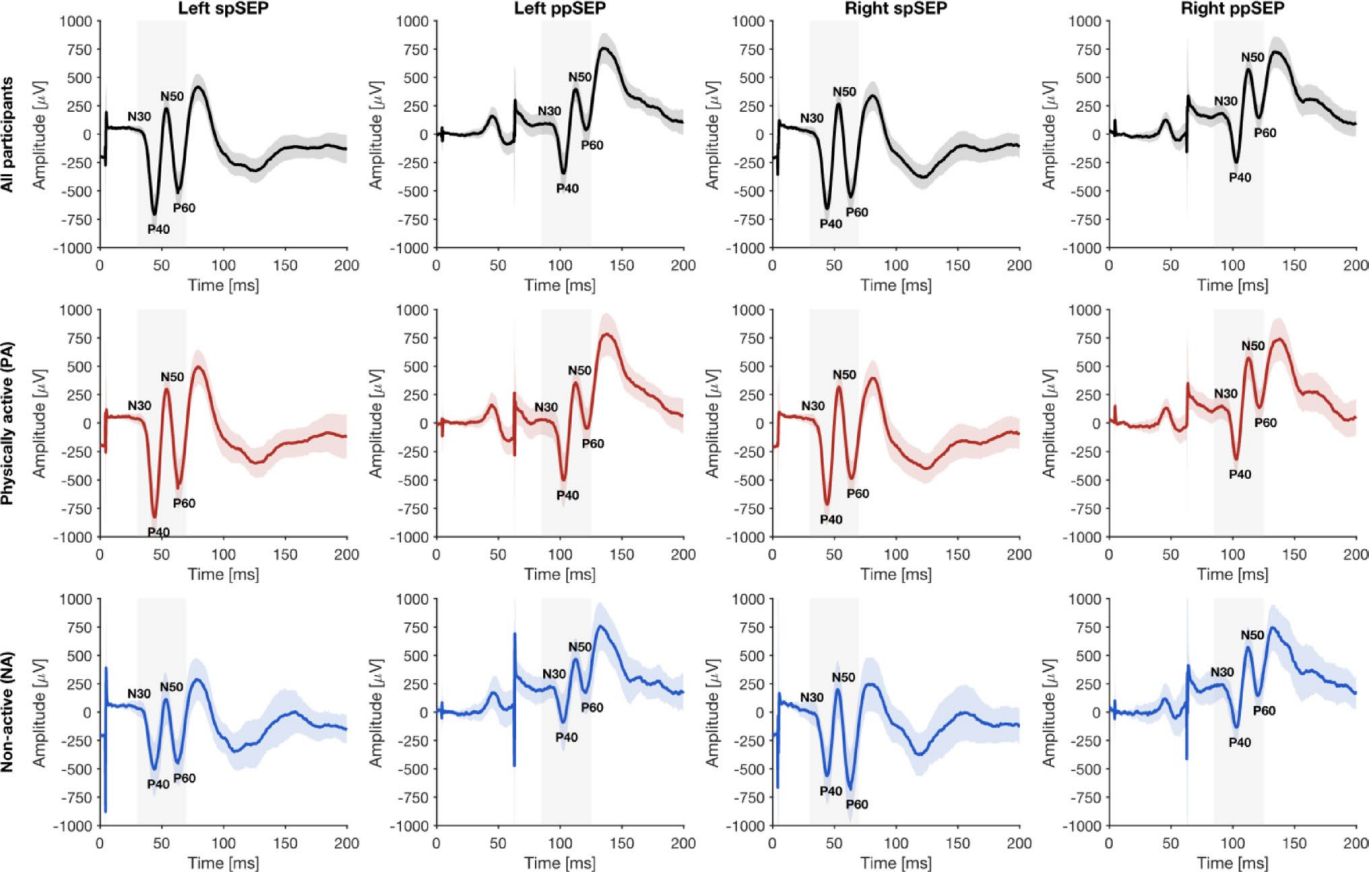
Grand average SEP waveforms with 95% confidence interval for left and right spSEPs and ppSEPs for all participants tested (n = 59) as well as for physically active (PA, n = 38) and non-active participants (NA, n = 21). Please note that for ppSEPs, all SEP amplitudes were suppressed as compared to spSEP indicating paired-pulse suppression (PPS)

For all participants tested, SEP amplitudes did not differ between right and left TN stimulation (N30/P40: *V* = 1179, *p*_*adj*_ = 0.080, *r* = 0.33; P40/N50: *V* = 966, *p*_*adj*_ = 0.695, *r* = 0.13 and N50/P60*: V* = 760, *p*_*adj*_ = 0.695, *r* = -0.14, see Fig. 1 for grand-average waveforms).

### spSEP amplitudes: comparison between PA and NA

spSEP amplitudes did not differ between PA and NA (*F*(1, 56.99) = 2.21, *p* = 0.143, η_p_^2^ = 0.04) or between the left or right leg (*F*(1, 284.01) = 1.91, *p* = 0.169, η_p_^2^ = 0.01). Furthermore, there was no significant interaction between groups (PA vs. NA) and TN stimulation (left vs. right TN) (*F*(1, 284.01) = 2.86, *p* = 0.092, η_p_^2^ = 0.01). A significant main effect of spSEP amplitudes (N30/P40, P40/N50 and N50/P60) was found (*F*(2, 284.01) = 15.61, *p* < 0.001, η_p_^2^ = 0.10). Interactions were not significant (group × spSEP amplitudes (*F*(2, 284.01) = 1.36, *p* = 0.258, η_p_^2^ = 0.01); TN stimulation × spSEP amplitudes (*F*(2, 284.01) = 1.15, *p* = 0.317, η_p_^2^ = 0.01). In addition, spearman rank correlations showed no significant associations between weekly physical activity and spSEP amplitudes for either left (N30/P40: ρ = -0.10, *p*_*adj*_ = 1.000; P40/N50: ρ = − 0.10, *p*_*adj*_ = 1.000; N50/P60: ρ = − 0.22, *p*_*adj*_ = 0.556 or right TN stimulation (N30/P40: ρ = − 0.11, *p*_*adj*_ = 1.000; P40/N50: ρ = − 0.01, *p*_*adj*_ = 1.000; N50/P60: ρ = 0.06, *p*_*adj*_ = 1.000; for details see Table 1).

**Table 1:** SEP amplitudes and corresponding PPS after right and left TN stimulation for all participants tested (n = 59; values are expressed in microvolts (µV) as mean ± SD)

		N30/P40	P40/N50	N50/P60
Right TN stimulation	spSEP amplitude	2.35 ± 1.62	3.32 ± 2.54	3.00 ± 1.90
	PPS (ratio)	0.73 ± 0.40	0.97 ± 0.56	0.59 ± 0.34
Left TN stimulation	spSEP amplitude	2.70 ± 1.84	3.58 ± 2.63	2.98 ± 2.14
	PPS (ratio)	0.68 ± 0.43	0.87 ± 0.76	0.59 ± 0.42

### ppSEP suppression (PPS)

**Fig. 2:**
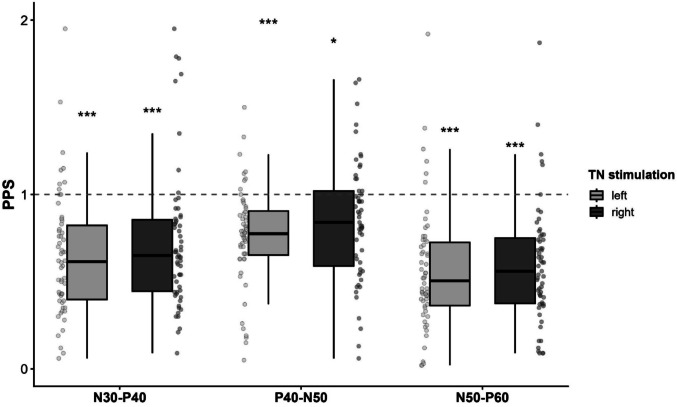
Overall paired-pulse suppression (PPS) after left and right TN stimulation across SEP amplitudes. The dashed horizontal line denotes the reference value of 1. Individual data points represent single-subject values. Asterisks indicate significant suppression relative to 1 (*p < .05, **p < .01, ***p < .001)

For all participants tested, we observed significant PPS for all SEP amplitudes tested after both left (N30/P40: *W* = 184, *p*_*adj*_ < 0.001, *r* = − 0.78; P40/N50: *W* = 264.5, *p*_*adj*_ < 0.001, *r* = − 0.68; N50/P60: *W* = 154.5, *p*_*adj*_ < 0.001, *r* = − 0.83) and right TN stimulation (N30/P40: *W* = 302.5, *p*_*adj*_ < 0.001, *r* = − 0.66; P40/N50: *W* = 601.5, *p*_*adj*_ = 0.025, *r* = − 0.30; N50/P60: *W* = 100, *p*_*adj*_ < 0.001, *r* = − 0.88; for details see Table 1 and Fig. 1 and 2).

### PPS: comparison between PA and NA

**Fig. 3:**
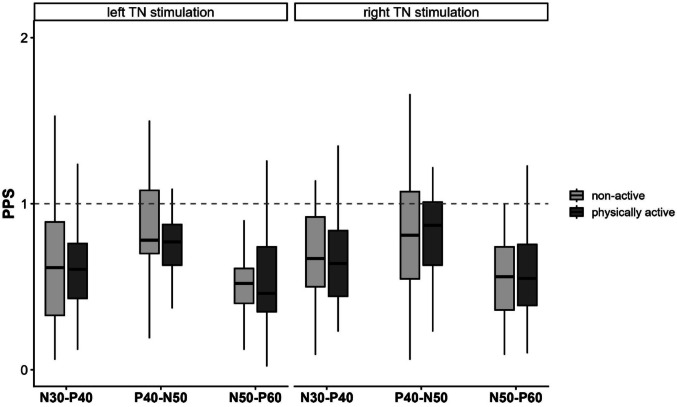
PPS across SEP amplitudes between physically active (PA) and non-active (NA) participants. Panels separate left and right TN stimulation. The dashed horizontal line denotes the reference value of 1 (no PPS)

There was no significant main effect for factor group (PA vs. NA (*F*(1, 56.91) = 0.27, *p* = 0.608, η_p_^2^ = 0.00) and TN stimulation (*F*(1, 284.10) = 2.31, *p* = 0.129, η_p_^2^ = 0.01). The group × TN stimulation interaction was also not significant (*F*(1, 284.10) = 0.19, *p* = 0.661, η_p_^2^ < 0.001). A significant main effect of ppSEP amplitudes was observed (*F*(2, 284.11) = 23.87, *p* < 0.001, η_p_^2^ = 0.14). Interactions between ppSEP amplitudes, group and TN stimulation were not significant (group × ppSEP amplitudes (*F*(2, 284.10) = 0.36, *p* = 0.698, η_p_^2^ = 0.00); TN stimulation × ppSEP amplitudes (*F*(2, 284.10) = 0.17, *p* = 0.845, η_p_^2^ = 0.00) and group × TN stimulation × ppSEP amplitudes (*F*(2, 284.10) = 0.58, *p* = 0.560, η_p_^2^ = 0.00, see Fig. 3).

**Fig. 4:**
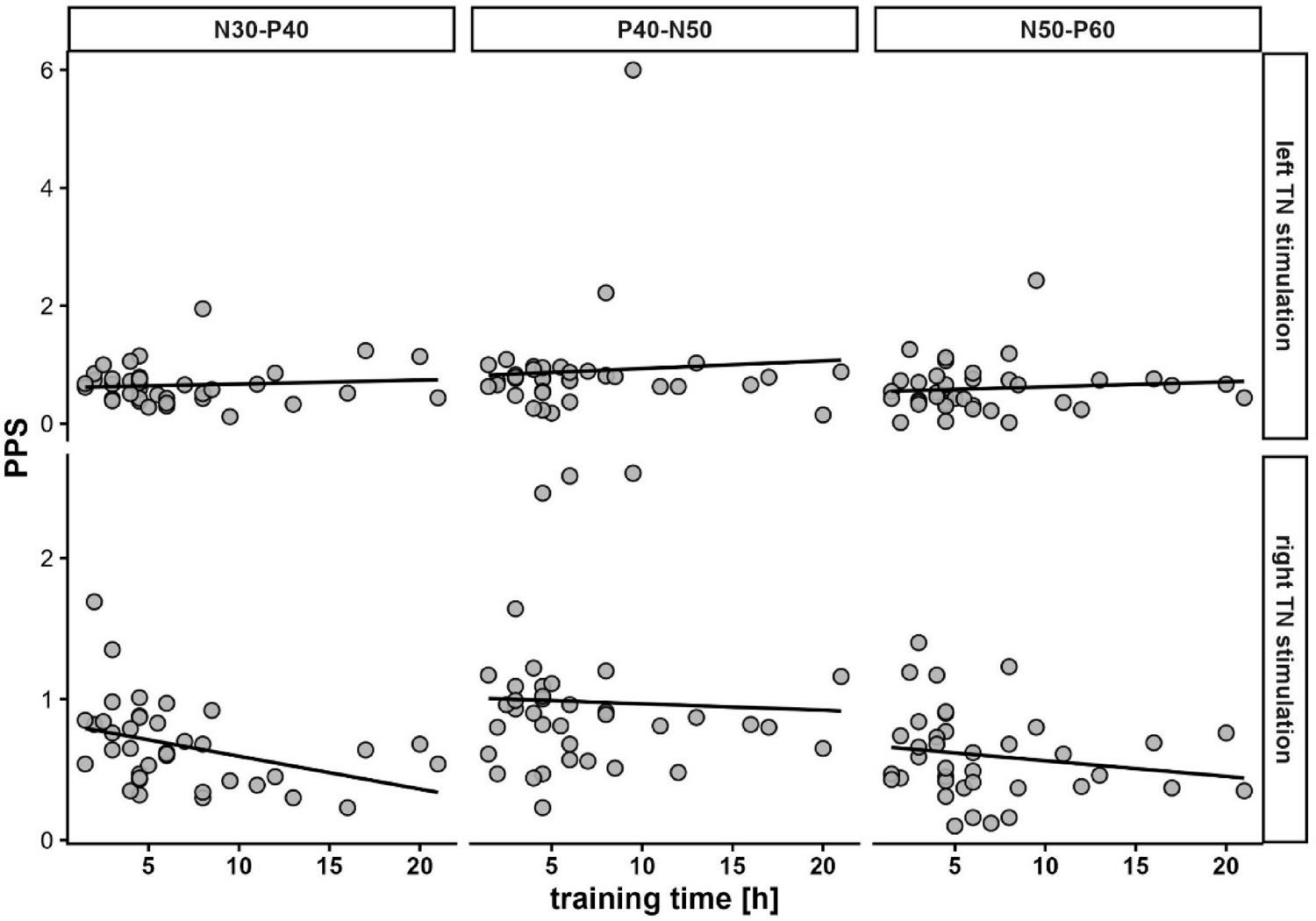
Correlations between training time and PPS across ppSEP amplitudes for PA. Solid lines depict the fitted linear regression for each panel. Please note, that there was only a significant association between the N30/P40 amplitude and the training time for right TN stimulation (ρ = − 0.46, p_adj_ = 0.012)

Interestingly, a spearman rank correlation in PA showed a negative association between PPS of the N30/P40 and the amount of physical activity per week for right TN stimulation (ρ = − 0.46, *p*_*adj*_ = 0.012), whereas no such association could be observed for the other ppSEP amplitudes (P40/N50: ρ = − 0.08, *p*_*adj*_ = 0.617; N50/P60: ρ = − 0.29, *p*_*adj*_ = 0.152). For left TN stimulation, no significant associations could be observed (N30/P40: ρ = − 0.15, *p*_*adj*_ = 1.000; P40/N50: ρ = 0.04, *p*_*adj*_ = 1.000; N50/P60: ρ = 0.09, *p*_*adj*_ = 1.000; for details see Fig. 4).

## Discussion

The present study investigated differences in spSEP and ppSEP amplitudes after left and right TN stimulation in PA and NA participants. Contrary to our initial hypothesis, no significant group differences were observed between PA and NA across SEP conditions (spSEP and ppSEP). However, within PA, a significant association emerged between the amount of physical activity and PPS specifically in the N30/P40 amplitude after right TN stimulation. This relationship was not observed for other ppSEP amplitudes or after left TN stimulation. These findings suggest that regular physical activity is not associated with general alterations in somatosensory processing but may selectively influence paired-pulse inhibition in a dose-dependent manner.

Some electrophysiological studies in highly trained athletes have reported group differences in spSEP parameters compared to non-athletes, but the findings across the literature are quite heterogeneous (Maudrich et al. 2022). For example, a study comparing elite gymnasts, endurance runners and sedentary controls found no robust differences in spSEP amplitudes or peak latencies, although correlations with training variables were reported (Thomas and Mitchell 1996). These findings align with the absence of group differences between PA and NA in our study and highlights that regular physical activity may not be sufficient to alter early somatosensory processing and paired-pulse inhibition. However, Maudrich and colleagues (2022) reported that several studies do indeed observe differences in spSEP amplitudes and latencies between athletes and controls. The authors conclude that while SEP measures can reflect sport-related neuroplastic adaptations, these effects are modal- and task-specific and may depend on the specific training content, intensity and methodological consistency across studies. Several factors may have contributed to the lack of significant SEP differences in the current study:

1. Although PA maintained regular activity for at least 2 years, the weekly training volume showed a huge variability among participants ranging from ~ 1 to 20 h/week. In fact, the mean engagement in regular exercise was 6.8 ± 5 h/week in PA which is relatively modest compared to structured athletic training which has been reported in other SEP studies (for review see Maudrich et al. 2022). Many spSEP findings relate to highly trained populations with multiple training sessions per week and sport-specific sensorimotor demands that could drive neuroplastic adaptations that are detectable with SEP recordings. For ppSEPs, it has been shown that a single bout of moderate-intensity aerobic exercise is capable of modulating PPS immediately after exercise, without altering spSEP amplitudes (Yamazaki et al. 2020). These findings highlight the potential and specificity of acute aerobic exercise-induced effects in the somatosensory domain.
2. spSEP and ppSEP components reflect general somatosensory processing, which may be less sensitive to moderate regular physical activity compared to other brain areas. For example, neuroplastic adaptations to regular physical activity might be more prominent in motor networks or higher-order cognitive processes rather than in somatosensory pathways. For example, physical activity or regular exercise has been shown to modulate cortical excitability and intracortical inhibition within M1 as assessed by means of transcranial magnetic stimulation (Lulic et al. 2017). Using paired-pulse transcranial magnetic stimulation (ppTMS) protocols it has been shown that athletes and musicians exhibit a decreased intracortical and interhemispheric inhibition in M1 (Ridding et al. 2000; Nordstrom and Butler 2002; Shin et al. 2012; Mason et al. 2020; Akalu et al. 2024).
3. Heterogeneity within groups in terms of fitness levels, exercise type, and other lifestyle factors may have attenuated potential group differences. Likewise, inactive participants, even if not engaging regularly in structured sport, may still accumulate incidental, but more moderate physical activity that at least partially mitigates differences in SEP parameters. Taken together, a more homogeneous group of PA (e.g. one sport instead of multiple disciplines) might have yielded different results and should therefore be considered in future studies that try to disentangle the effect of physical activity especially on PPS in the somatosensory domain.

Apart from the lack of effect on spSEP and ppSEP measures between PA and NA, we showed that PPS in PA after right TN stimulation is significantly modulated by the amount of physical activity. More specifically, higher hours of training per week were accompanied by stronger PPS in early SEP components such as the N30/P40 amplitude. This SEP component seems to be a composite measure which most likely captures mixed subcortical and cortical contributions. The N30 is generally considered a far-field subcortical response of the dorsal column-medial lemniscus pathway, whereas the P40 reflects the first major cortical response generated in S1 (Urasaki et al. 1993; Kakigi et al. 1995; Baumgärtner et al. 1998). Comparable PPS effects were not observed for later cortical SEP components or after left TN stimulation. Taken together, these findings indicate that long-term physical training is associated with a selective modulation of early somatosensory processing, rather than a general alteration of ppSEP amplitudes. The fact that spSEPs were comparable between groups, while the PPI of the N30/P40 amplitude was affected by physical activity suggests that exercise predominantly alters the dynamic interaction between successive inputs (i.e., gating properties).

The finding of increased PPS of the N30/P40 amplitude in more trained individuals was contrary to our a priori hypothesis that increasing training exposure would reduce PPS. This hypothesis was based on previous studies using ppTMS protocols that showed decreased intracortical and interhemispheric inhibition in M1 of athletes (Mason et al. 2020; Akalu et al. 2024) and musicians (Ridding et al. 2000; Nordstrom and Butler 2002; Shin et al. 2012).

However, it is important to emphasize that ppTMS in M1 and pp SEPs in S1 index different neural circuits, different cortical areas, and different time scales of inhibitory processing. Therefore, opposite effects in these neurophysiological measures do not necessarily represent a contradiction but may instead reflect a redistribution of inhibitory control within the sensorimotor network, in which relatively disinhibited motor output coexists with more pronounced early sensory gating. This interpretation is also compatible with studies demonstrating acute disinhibition of S1 after a single bout of moderate (aerobic) exercise. For example, Yamazaki and colleagues reported reduced PPS and altered temporal discrimination thresholds after acute aerobic exercise, which the authors interpreted as a transient release of inhibition (Yamazaki et al. 2020). Crucially, this study examined short-term and state-dependent changes in ppSEPs at specific ISIs after median nerve stimulation, whereas the present work focused on differences between individuals with no or regular (> 1 h/ week) physical activity in the lower limb (TN). Thus, it is reasonable to assume that acute exercise temporarily reduces inhibitory tone in S1, while regular training over weeks, months or years may result in stronger paired-pulse inhibition as reflected by enhanced N30/P40 PPS. However, because the N30/P40 complex likely reflects contributions from both cortical and subcortical generators, the present findings should not be interpreted as evidence for a specifically cortical inhibitory mechanism.

The apparent discrepancy between findings in M1 and S1 suggests that training-related changes in inhibitory circuity in the somatosensory system could instead be associated with an upregulation of inhibitory control or efficiency, potentially supporting a more selective sensorimotor processing in physically active individuals. In support of this notion, pianists showed experience-dependent enhanced somatosensory inhibition which seems to be associated with optimized sensorimotor performance (Hirano et al. 2025). Similar findings have been described in non-primary motor areas of string players when compared to non-musicians (Vaalto et al. 2016). Extrapolating from these findings in musicians, one may speculate that comparable mechanisms operate in PA which may develop a more efficient neural filter that selectively attenuates predictable, repetitive, or task-irrelevant afferent input, thereby sharpening the representation of unexpected or behaviorally salient events. This kind of experience-dependent tuning would be particularly beneficial in sports that require continuous postural regulation, rapid responses to perturbations, and precise timing of lower-limb movements, where an overload of unfiltered sensory information could otherwise be detrimental (Attalin et al. 2024; Yılmaz et al. 2024).

While the neurophysiological and behavioral outcome of increased inhibition in S1 still remains elusive, it is tempting to speculate that regular exercise might enhance the ability to suppress irrelevant afferent inputs, thereby improving signal-to-noise ratio and sensory selection. However, the increase in PPS was only observed for the N30/P40 amplitude after right TN stimulation and should therefore be interpreted with caution since this SEP amplitude likely captures mixed generator contributions (subcortical and cortical).

Finally, several limitations of the present study must be acknowledged. First, the cross-sectional design precludes causal inferences. Hence, longitudinal designs, for example across structured exercise/ training interventions, would be necessary to disentangle if the amount of exercise is indeed capable of evoking alterations in PPS and if similar effects could be observed using different ISIs. Additionally, the sample size, although comparable to previous SEP studies, may have limited statistical power and correlation-based approaches. Therefore, it is possible that only one significant correlation (PPS of the N30/P40 and the amount of physical activity per week for right TN stimulation) was detected. However, most of the remaining non-significant correlations showed only small or negligible effect sizes. Thus, their non-significant results are less likely to be explained by type II errors, although this possibility cannot be completely excluded.

Future studies with especially more homogeneous cohorts would help to better characterize the robustness and generalizability of the observed effects.

Furthermore, on the day of SEP measures, we did not control for potentially relevant confounders such as prior sleep duration or quality, acute fatigue, time since last training session, or habitual physical activity outside the primary sport. Given evidence that acute aerobic exercise can modulate PPS in S1, interindividual variability in physical activity (e.g., timing and intensity of the last training session) shortly before SEP recordings could have influenced the amount of individual PPS. Finally, the classification into PA and NA was based on self-reported weekly training hours, which may be subject to recall bias and does not capture qualitative aspects of training (e.g., type of sport, proportion of technical vs. conditioning work, or balance versus strength emphasis).

Moreover, linking PPS after TN stimulation to behavioral parameters such as balance control, postural stability or force generation of the lower limb would help to clarify the functional significance of PPS in S1. Multimodal approaches including TMS measures of M1 excitability and inhibition could further elucidate how PPS in S1 relates to motor cortical plasticity and whether distinct profiles of sensorimotor inhibition characterize different types of sports.

## Data Availability

No datasets were generated or analysed during the current study.
